# Supplementary material for: EXO1 promotes the meiotic MLH1-MLH3 endonuclease through conserved interactions with MLH1, MSH4 and DNA
Source: Nat Commun. 2025 May 3;16:4141. doi: 10.1038/s41467-025-59470-2 (PMC12049449; doi:10.1038/s41467-025-59470-2)
Supplement: Supplementary file 1 — Supplementary Information [file 41467_2025_59470_MOESM1_ESM.pdf]

Supplemental information for:

EXO1 promotes the meiotic MLH1-MLH3 endonuclease through conserved interactions with MLH1, MSH4 and DNA

Megha Roy[1#], Aurore Sanchez[1#§]*, Raphael Guerois[2], Issam Senoussi[1,3], Arianna Cerana[4], Jacopo Sgrignani[1], Andrea Cavalli[1], Andrea Rinaldi[4] and Petr Cejka[1]*

Affiliations

[1]Institute for Research in Biomedicine, Università della Svizzera italiana (USI), Faculty of Biomedical Sciences, Bellinzona, 6500, Switzerland

[2]Université Paris-Saclay, CEA, CNRS, Institute for Integrative Biology of the Cell (I2BC), 91198, Gif-sur-Yvette, France.

[3]Department of Biology, Institute of Biochemistry, Eidgenössische Technische Hochschule (ETH), Zürich, 8093, Switzerland

[4]Institute of Oncology Research, Università della Svizzera italiana (USI), Faculty of Biomedical Sciences, 6500 Bellinzona, Switzerland.

[#]M. Roy and A. Sanchez contributed equally to this study.

[§]Present address: Institute Curie, 26 rue d'Ulm, 75248 Paris Cedex, France

*Correspondence: aurore.sanchez@curie.fr , petr.cejka@irb.usi.ch

Table of contents:

Supplementary Figures 1-7 and their legends

Supplementary Table 1: Oligonucleotides used in this study.

Supplementary Table 2: Description of conditions used to generate models.

Supplementary Table 3: Summary of evaluation scores for the model generated with the highest confidence score for all the models generated by AlphaFold2

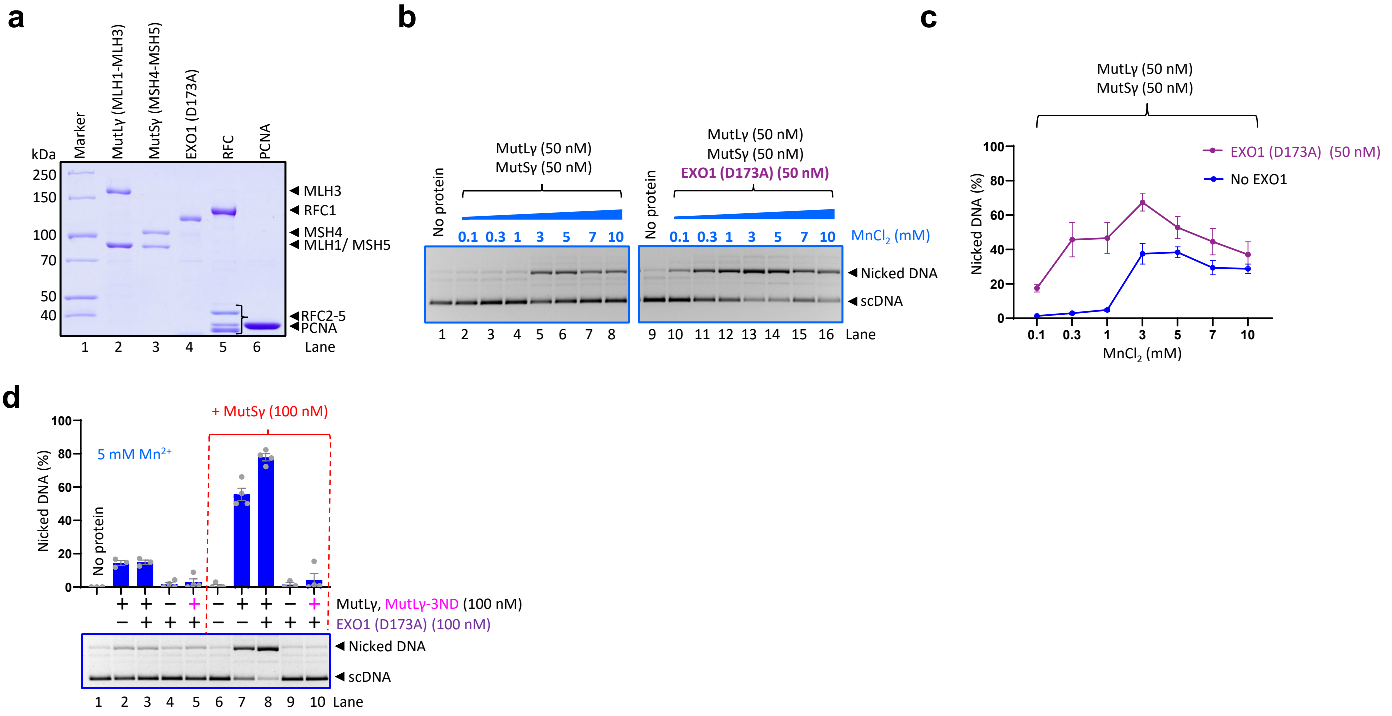

Supplementary Figure 1. DNA cleavage by MutLγ and MutSγ and its stimulation by EXO1 (D173A).

(**a**) Recombinant proteins used in this study. The polyacrylamide gel was stained with Coomassie brilliant blue. A Representative of two independent experiments

(**b**) Nicking assays with MutLγ and MutSγ in the presence of increasing concentrations of manganese. The reaction was carried out in the absence (left) or in the presence (right) of EXO1 (D173A). A representative gel of three independent experiments.

(**c**) Quantification of experiments such as in B. Averages shown, n = 3 independent experiments; error bars, SEM.

(**d**) Nicking assays to test the effect of MutSγ and/or EXO1 (D173A) on the stimulation of MutLγ in the presence of 5 mM Mn^{2+}. Nuclease dead MutLγ (MutLγ-3ND, D1223N, Q1224K, E1229K) was used as a negative control. Top, quantification; averages shown, n = 3 for lanes 2, 3, and 9, n = 4 for all other samples (independent experiments); error bars, SEM. Bottom, a representative gel. Source data are provided as a Source Data file.

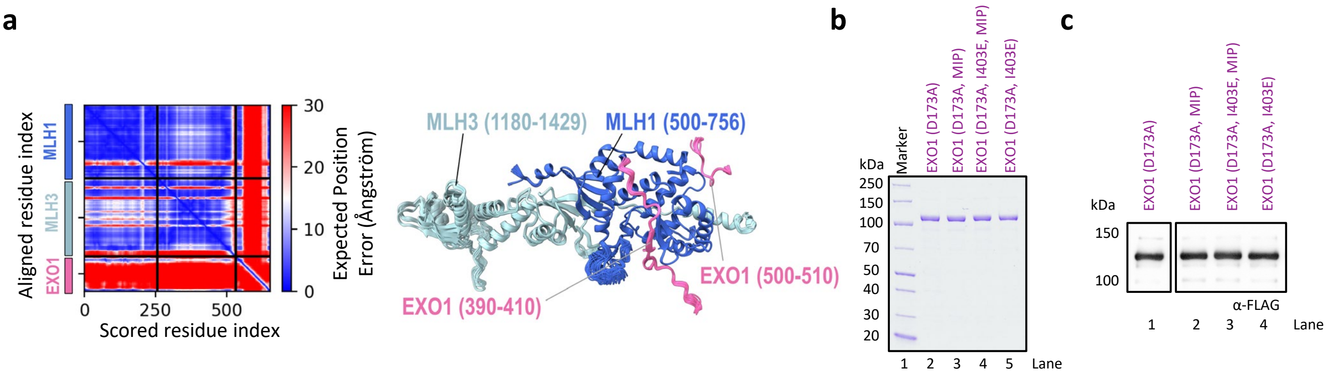

Supplementary Figure 2. Interaction of EXO1 and MutLγ.

(**a**) Characteristics of the structural model of the complex between MLH1 (500-756), MLH3 (1180-1429) and EXO1 (390-510). Left, predicted alignment error plot generated by AlphaFold2 for the model with the highest confidence score. Right, superposition of the 25 models generated by AlphaFold2.

(**b**) Recombinant EXO1 mutants used in this study as depicted in Fig. 2C. F506A and F507A mutations within the MIP motif are referred to as the MIP mutations. A representative of three polyacrylamide gel stained with Coomassie brilliant blue.

(**c**) Western blot of EXO1 variants used in Fig. 3D. 100 ng of the recombinant proteins were loaded and detected using anti-FLAG antibody. F506A and F507A mutations within the MIP motif are referred to as the MIP mutations. The antibody recognizes all EXO1 variants in the same way. Source data are provided as a Source Data file.

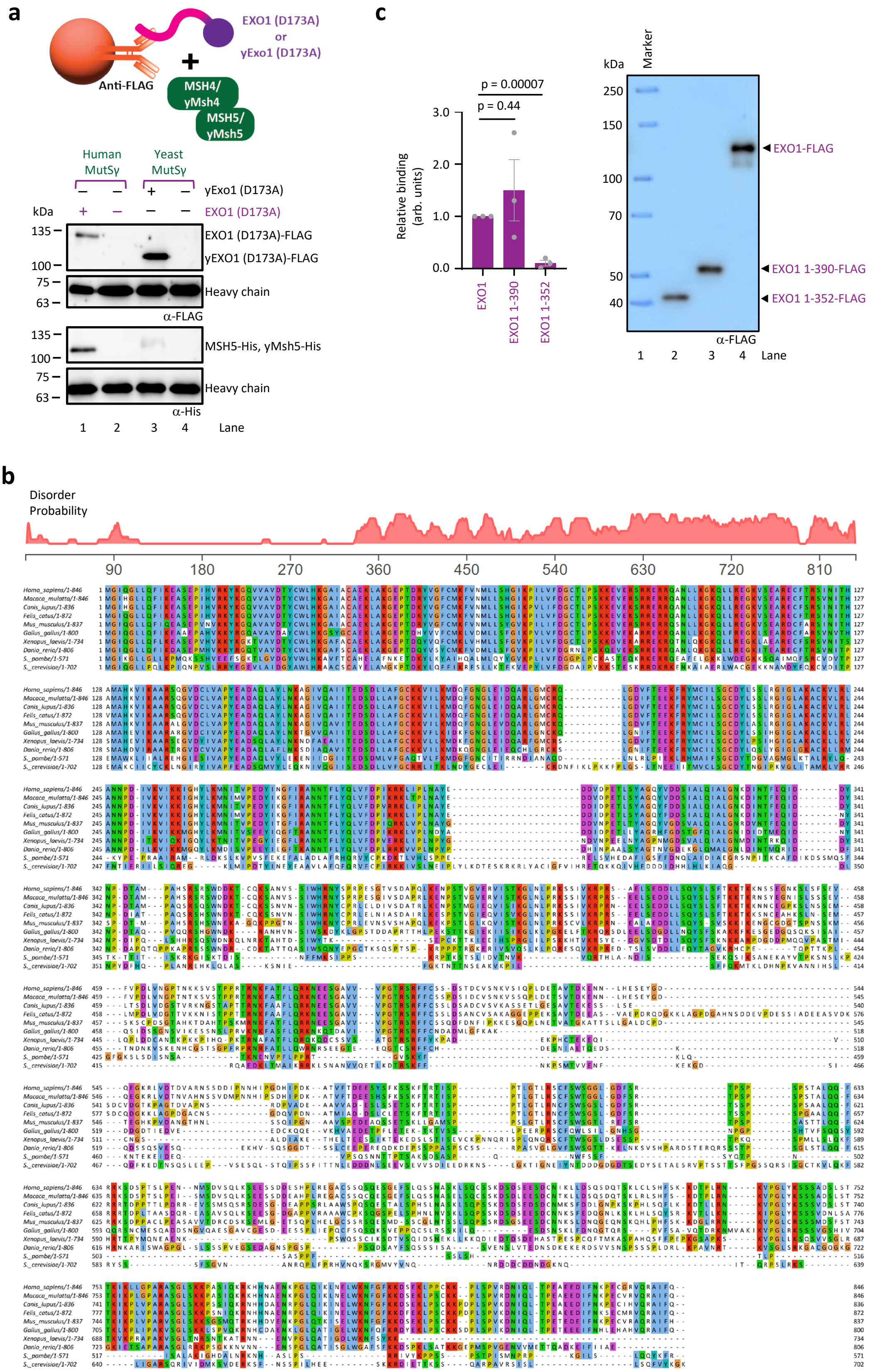

Supplementary Figure 3. Interaction of EXO1 and MutSγ.

(**a**) Protein interaction assays of MutSγ and EXO1 (D173A) or yeast MutSγ and yeast Exo1 (D173A). EXO1 (D173A)-FLAG or yeast Exo1 (D173A)-FLAG (bait) were immobilized using anti-FLAG resin, and human MutSγ (MSH4-Strep-MSH5-His), or yeast MutSγ (Msh4-Strep-Msh5-His) (prey) were subsequently added. A representative of three and two independent experiments with human and yeast proteins respectively is shown, as analyzed by Western blotting.

(**b**) Illustration from MobiDB-lite showing the predicted disorder probability of EXO1 (UniProt Id: Q9UQ84) in orange. The scale represents the amino-acid position. Below, multiple sequence alignment of EXO1 across different species generated using the MAFFT method and represented using Jalview.

(**c**) Left, quantification of interaction assays of MutSγ (bait) and EXO1 variants (prey) as in Fig. 3h. The signal was normalized to EXO1, averages shown, n = 3 independent experiments; error bars, SEM; two-tailed t-test. Right, western blot of EXO1 truncation variants used in Fig. 3h. 100 ng of the recombinant proteins were loaded and detected using anti-FLAG antibody. The membrane was stained with Coomassie brilliant blue, and the image is an overlay of the signal and the marker. Source data are provided as a Source Data file.

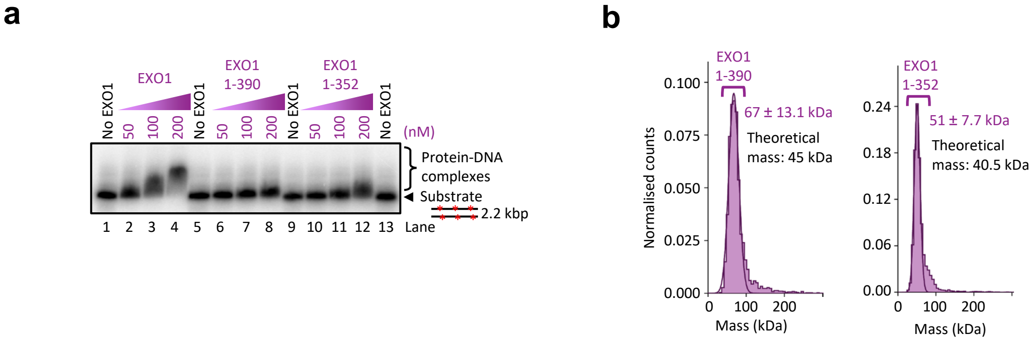

Supplementary Figure 4. Analysis of full length EXO1 and truncation variants.

(**a**) Electrophoretic mobility shift assays using randomly labeled 2.2 kbp-long dsDNA with recombinant EXO1, EXO1 1-390, and EXO1 1-352 as indicated. The red asterisks indicate the radioactive labels. Shown is a representative gel of four independent experiments.

(**b**) Molecular weight distributions of EXO1 1-390-FLAG (theoretical mass 45 kDa) and EXO1 1-352-FLAG (theoretical mass 40.5 kDa) as measured by mass photometry. The measured mass is reported as median ± standard deviation. Source data are provided as a Source Data file.

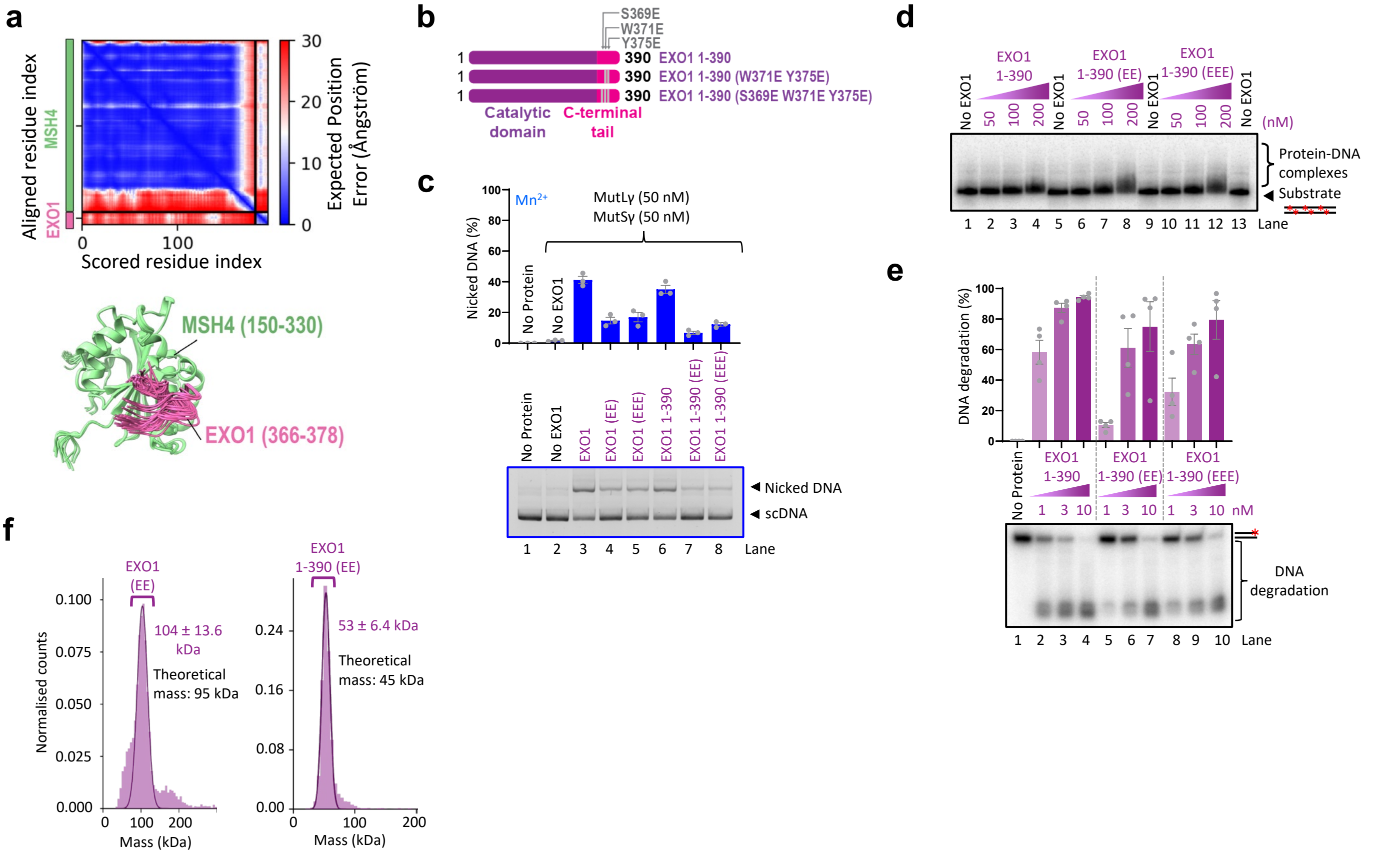

Supplementary Figure 5. Analysis of EXO1 variants deficient in the stimulation of MutSγ-MutLγ.

(**a**) Characteristics of the structural model of the complex between MSH4 (150-330), and EXO1 (366-378). Top, a predicted alignment error plot generated by AlphaFold2 for the model with the highest confidence score. Bottom, superposition of the 25 models generated by AlphaFold2**.**

(**b**) Schematic of the EXO1 1-390 point mutants used in this study. Recombinant EXO1 1-390 (W371E Y375E) is abbreviated as EXO1 1-390 (EE) and EXO1 1-390 (S369E W371E Y375E) is abbreviated as EXO1 1-390 (EEE).

(**c**) Stimulation of MutLγ-MutSγ complex nicking activity by the indicated EXO1 variants in the presence of 0.6 mM Mn^{2+}. Top, quantification; averages shown, n = 3 independent experiments; error bars, SEM. Bottom, a representative gel.

(**d**) Electrophoretic mobility shift assays with recombinant EXO1 1-390, EXO1 1-390 (EE) and EXO1 1-390 (EEE) as indicated, using randomly labeled 2.2 kbp-long dsDNA as a substrate. The red asterisks indicate the positions of the radioactive labels. Shown is a representative of four independent experiments.

(**e**) Nuclease assays using 3'-labeled 50 bp dsDNA with indicated EXO1 variants. Top, quantification. Averages shown, n = 4 independent experiments; error bars, SEM. Bottom, a representative gel. The red asterisk indicates the position of the radioactive label.

(**f**) Molecular weight distributions of EXO1-EE-FLAG, EXO1-EEE-FLAG (theoretic mass 95 kDa), EXO1 1-390-EE-FLAG, and EXO1 1-390-EEE-FLAG (theoretical mass 45 kDa) as measured by mass photometry. The measured mass is reported as median ± standard deviation. Source data are provided as a Source Data file.

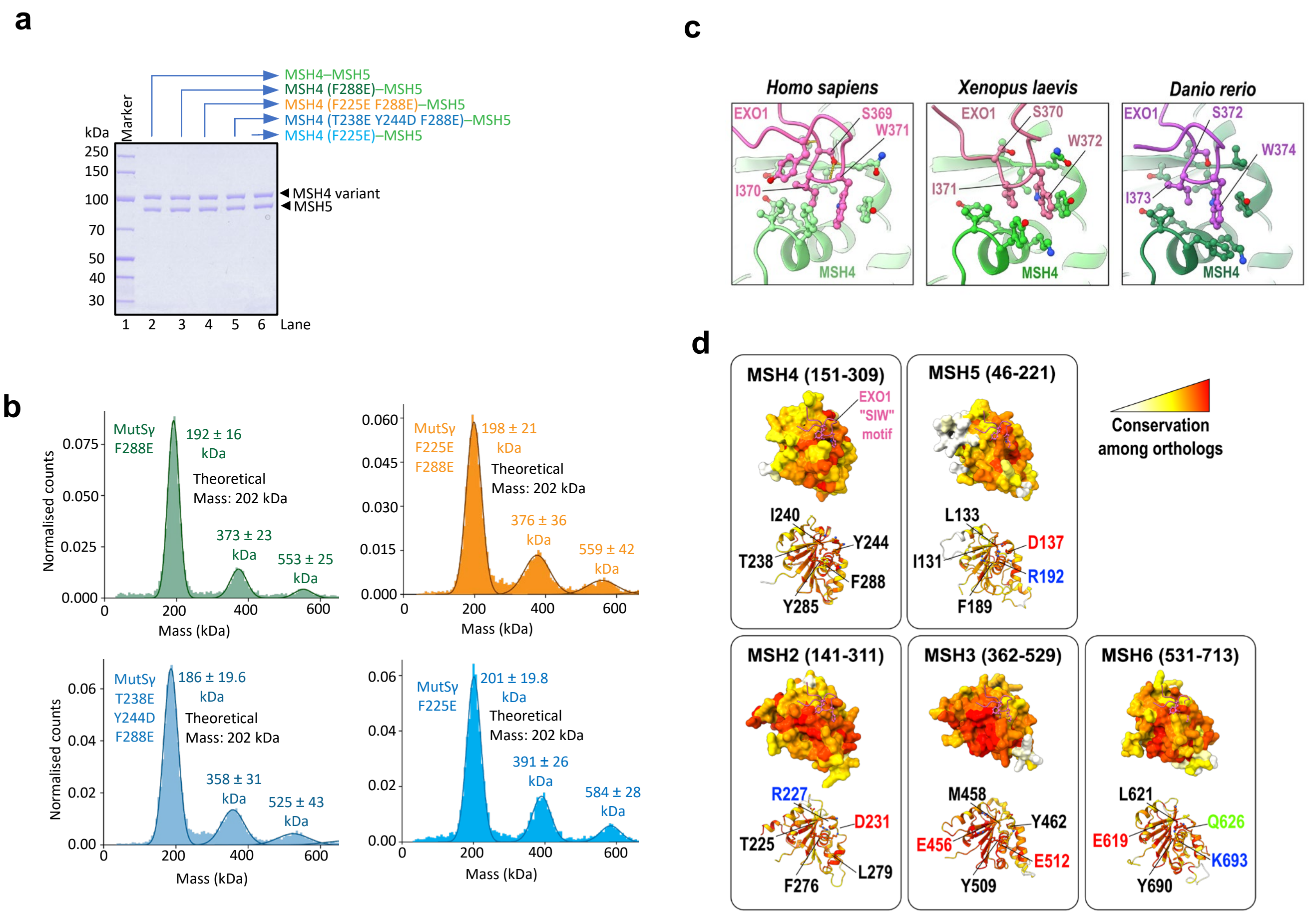

Supplementary Figure 6. Interaction of MSH4 and EXO1.

(**a**) A representative of two polyacrylamide gel showing MutSγ variants with mutations in MSH4. The polyacrylamide gel was stained with Coomassie Brilliant Blue.

(**b**) Molecular weight distributions of MutSγ variants (theoretical mass 202 kDa) as measured by mass photometry. The measured mass is reported as median ± standard deviation.

(**c**) Comparative structural analysis of the predicted interface between MSH4 and EXO1 in other vertebrates. The interface between the connector domain of MSH4 (green) and the SIW motif in EXO1 (pink) was modelled using AlphaFold2 for two additional vertebrate species *Xenopus laevis* and *Danio rerio*. The residues involved in the interface are highlighted as ball-and-stick representation. The residues of the SIW motif in EXO1 are invariant in *Homo sapiens*, *Xenopus laevis* and *Danio rerio,* while the human Y375 is not conserved in the other species, consistent with the mutagenesis analysis. On the MSH4 side, most of the observed substitutions are conservative and the major hydrophobic residues are conserved.

(**d**) Comparative analysis of the connector domains in MutS family proteins homologous to MSH4 in humans. The connector domains of the five MutS proteins are represented to highlight the amino acids and the conservation of the region that, in MSH4, is involved in binding to the SIW motif of EXO1. In each panel, a surface representation of the domain is colored according to the conservation index. The region where the SIW motif of EXO1 binds to the domain is identified in the surface representation by the peptide model, as modeled in interaction with MSH4. The side chains of the amino acids involved in the interaction are shown in a ball-and-stick representation, with the five key residues labeled. The label color corresponds to the nature of the amino acids: black for hydrophobic and weakly polar, red for acidic, blue for basic, and green for highly polar residues. The boundaries of the connector domains in each homolog are indicated in the title of each panel. Source data are provided as a Source Data file.

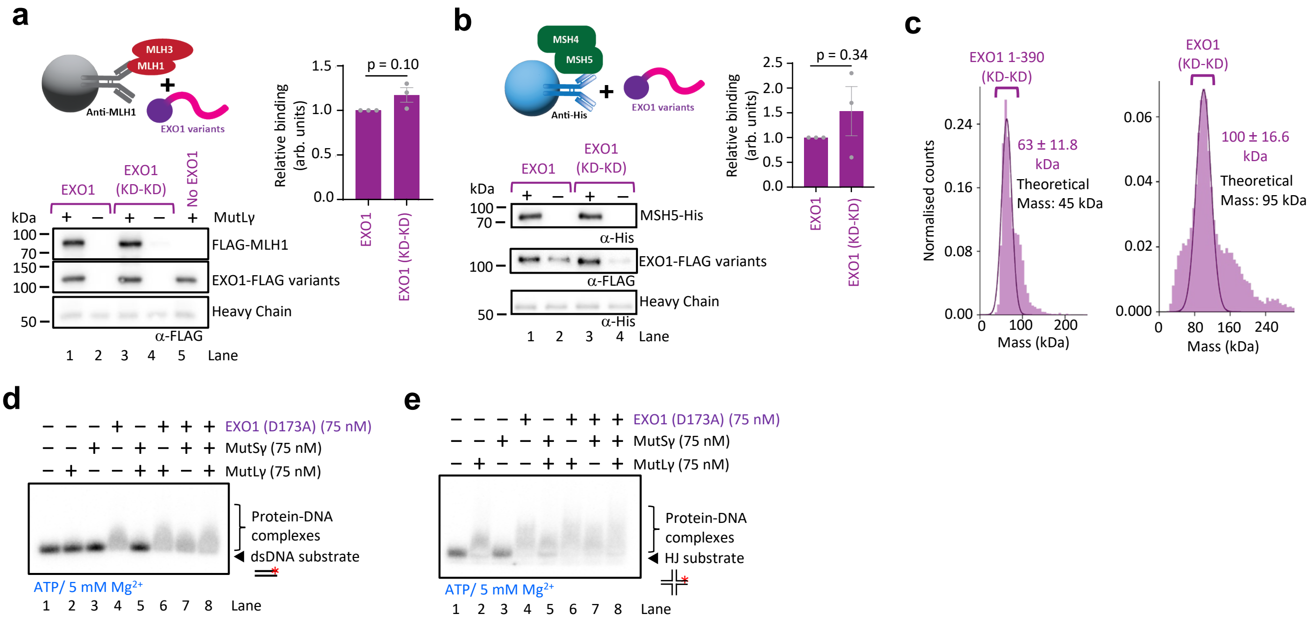

Supplementary Figure 7. Analysis of DNA binding by EXO1

(**a**) Protein interaction assays of MutLγ and EXO1 variants. MutLγ (FLAG-MLH1-MBP-MLH3) (bait) were immobilized using anti-MLH1 antibodies. EXO1 variants (preys) were subsequently added. Top, a schematic of the assays. Bottom, representative experiments as analyzed by Western blotting. Right, quantification of interaction relative to EXO1; averages shown, n = 3 independent experiments; error bars, SEM; two-tailed t-test.

(**b**) Protein interaction assays of MutSγ and EXO1 variants. MutSγ (MSH4-Strep-MSH5-His) (bait) were immobilized using anti-His antibodies. EXO1 variants (preys) were subsequently added. Top, a schematic of the assays. Bottom, representative experiments as analyzed by Western blotting. Right, quantification of interaction relative to EXO1; averages shown, n = 3 independent experiments; error bars, SEM; two-tailed t-test.

(**c**) Molecular weight distributions of EXO1-KD-KD-FLAG (theoretical mass 95 kDa) and EXO1 1-390-KD-KD-FLAG (theoretical mass 45 kDa) as measured by mass photometry. The measured mass is reported as median ± standard deviation.

(**d**) Electrophoretic mobility shift assays with recombinant proteins as indicated, using 3'-labeled 50 bp dsDNA. The red asterisks indicate the radioactive label. Shown is a representative of two independent experiments.

(**e**) Electrophoretic mobility shift assays with recombinant proteins as indicated, using 3'-labeled oligo-based HJ. The red asterisks indicate the radioactive label. Shown is a representative of two independent experiments. Source data are provided as a Source Data file.

Supplementary Table 1:
List of oligonucleotides used for the production of recombinant proteins.

Name	Sequence 5' to 3'
AS109 hMSH4-F288E For	CCCTGCTGAAGTACGTTGAAGAAATCCAGAACTCTGTCTACGC
AS110 hMSH4-F288E Rev	GCGTAGACAGAGTTCTGGATTTCTTCAACGTACTTCAGCAGGG
M11 hMSH4-Y244D For	CACCACGATTCAGCGCAAGGATTTTAATGAAACCAAAGG
M12 hMSH4-Y244D Rev	CCTTTGGTTTCATTAAAATCCTTGCGCTGAATCGTGGTG
M15 hMSH4-T238E-Y244D For	CGTGAATTTCGAAACGATTCAGCGCAAGGATTTTAATGAAACCAAAGGCCTGG
M16 hMSH4-T238E-Y244D Rev	CCAGGCCTTTGGTTTCATTAAAATCCTTGCGCTGAATCGTTTCGAAATTCACG
M17 hMSH4-F225E For	GGTAATAGCACGAAGCTGGAGACCCTGATCACG
M18 hMSH4-F225 Rev	CGTGATCAGGGTCTCCAGCTTCGTGCTATTACC
HEXO1_MIMFO primer	GTGGTTCCAGGGACCAGAAGCAGGGCTGCTTGCAGTTCAGATTCTAC
HEXO1_MIMRE primer	GTAGAATCTGAACTGCAAGCAGCCCTGCTTCTGGTCCCTGGAACCAC
AS92 hEXO1-I403E For	CTGTGGGAGTGGAACGAGTGGAAAGTACTAAAGGGTTAAATCTC
AS93 hEXO1-I403E Rev	GAGATTTAACCCTTTAGTACTTTCCACTCGTTCCACTCCCACAG
AS105 hEXO1-W371E-Y375E For	CAGCTAATGTTAGCAGCATTGAACATAGGAATGAATCTCCCAGACCAGAGTCGGG
AS106 hEXO1-W371E-Y375E Rev	CCCGACTCTGGTCTGGGAGATTCATTCCTATGTTCAATGCTGCTAACATTAGCTG
AS107 hEXO1-S369E-W371E-Y375E For	CAAAAGTCAGCTAATGTTAGCGAGATTGAACATAGGAATGAATCTCCCAGACCAGAGTCGGG
AS108 hEXO1-S369E-W371E-Y375E Rev	CCCGACTCTGGTCTGGGAGATTCATTCCTATGTTCAATCTCGCTAACATTAGCTGACTTTTG
M19 hEXO1-W371E For	CAGCTAATGTTAGCAGCATTGAACATAGGAATTACTCTCCCAGACCAGAGTCGGG
M24 hEXO1-W371E Rev	ACTCTGGTCTGGGAGAGTAATTCCTATGTTCAATGCTGCTAACATTAGCTG
M20 hEXO1-Y375E For	CAGCTAATGTTAGCAGCATTTGGCATAGGAATGAATCTCCCAGACCAGAGTCGGG
M25 hEXO1-Y375E Rev	ACTCTGGTCTGGGAGATTCATTCCTATGCCAAATGCTGCTAA
M21 hEXO1- W371A For	CAGCTAATGTTAGCAGCATTGCACATAGGAATTACTCTCCCAGACCAGAGTCGGG
M26 hEXO1- W371A Rev	TCTGGGAGAGTAATTCCTATGTGCAATGCTGCTAA
M22 hEXO1-Y375A For	CAGCTAATGTTAGCAGCATTTGGCATAGGAATGCATCTCCCAGACCAGAGTCGGG
M27 hEXO1-Y375A Rev	TGGTCTGGGAGATGCATTCCTATGCCAAAT
M23 hEXO1-W371A-W375A For	CAGCTAATGTTAGCAGCATTGCACATAGGAATGCATCTCCCAGACCAGAGTCGGG
M28 hEXO1-W371A-W375A Rev	TGGTCTGGGAGATGCATTCCTATGTGCAATGCTGCTAA
AS28 hEXO1-D78A For	TCAAGCCTATTCTCGTATTTGCTGGATGTACTTTACCTTCTAAAAAG
AS29 hEXO1-D78A Rev	CTTTTTAGAAGGTAAAGTACATCCAGCAAATACGAGAATAGGCTTGA
AS88 hEXO1-K185D For	GCTGTAAAAAGGTAATTTTAGACATGGACCAGTTTGGAAATGG
AS89 hEXO1-K185D Rev	CCATTTCCAAACTGGTCCATGTCTAAAATTACCTTTTTACAGC
AS90 hEXO1-K237D For	CTGCGTGGGATTGGATTAGCAGACGCATGCAAAGTCCTAAGACTAG
AS91 hEXO1-K237D Rev	CTAGTCTTAGGACTTTGCATGCGTCTGCTAATCCAATCCCACGCAG
AS86 hEXO1 DA-WT conversion For	CCATAATTACAGAGGACTCGGATCTCCTAGCTTTTGGCTGTAAAAAGG
AS87 hEXO1 DA-WT conversion Rev	CCTTTTTACAGCCAAAAGCTAGGAGATCCGAGTCCTCTGTAATTATGG
HEXO1FO_NHE	GCGCGCGCTAGCATGGGGATACAGGGATTGC
AS21 EXO1-1-787	CGCGCGCCCGGGCTACTTGTCGTCATCGTCTTTGTAGTCTAACCCCGGCTTGTTCTCGGC
AS22 EXO1-1-603	CGCGCGCCCGGGCTACTTGTCGTCATCGTCTTTGTAGTCTCCCAAAGTGGGTGGTGAAAT
AS23 EXO1-1-490	CGCGCGCCCGGGCTACTTGTCGTCATCGTCTTTGTAGTCTTTCCTTTGTAAAAATGTTGC
AS33 EXO1-1-390	CGCGCGCCCGGGCTACTTGTCGTCATCGTCTTTGTAGTCCAATTGTGGGGCATCTGAAAC
AS34 EXO1-1-352	CGCGCGCCCGGGCTACTTGTCGTCATCGTCTTTGTAGTCTCTTGAATGGGCAGGCATAG
M1 BamH1-EXO1 For	ATGCATGGATCCATGGGGATACAGGGATTGCTACAAT
M2 Xma1-FLAG-EXO1 Rev	CTCGAGCCCGGGCTACTTGTCGTCATCGT
NHEJ-FOR-BIO	(BIO) GTGCATGACTGCAGGTCGACTCTAGAGG
NHEJ-REV-NheI	TGCAGTTCGCTAGCCAGCTGCATTAATGAATCGG

Supplementary Table 2: Description of conditions used to generate models.

Index	Model name	Species	Subunits	Uniprot	Start	Stop	Size	Nb sequences
**1**	**MLH1-MLH3-EXO1**	*H. sapiens*	*Hs*MLH1	P40692	500	756	628	p=625,u=2609
			*Hs*MLH3	Q9UHC1	1180	1429		
			*Hs*EXO1	Q9UQ84	390	510		
**2**	**MSH4_EXO1**	*H. sapiens*	*Hs*MSH4	O15457	150	330	194	p=653, u=1230
			*Hs*EXO1	Q9UQ84	365	378		

Supplementary Table 3: Summary of evaluation scores for the model generated with the highest confidence score for all the models generated by AlphaFold2.

Index	Model name	Species	pLDDT	pTMscore	ipTMscore	Confidence score	PAE	Model Archive
**1**	**MLH1-MLH3-EXO1**	*H. sapiens*	80.82	0.77	0.70	0.716	Supplementary Fig. 2a	ma-m1f7g
**2**	**MSH4_EXO1**	*H. sapiens*	83	0.793	0.349	0.438	Supplementary Fig. 5a	ma-v510n
