# Supplementary material for: EXO1 promotes the meiotic MLH1-MLH3 endonuclease through conserved interactions with MLH1, MSH4 and DNA
Source: Nat Commun. 2025 May 3;16:4141. doi: 10.1038/s41467-025-59470-2 (PMC12049449; doi:10.1038/s41467-025-59470-2)
Supplement: Supplementary file 2 — Reporting Summary [file 41467_2025_59470_MOESM2_ESM.pdf]

nature portfolio

Corresponding author(s): Aurore Sanchez, Petr Cejka

Last updated by author(s): Apr 10, 2025

Reporting Summary

Nature Portfolio wishes to improve the reproducibility of the work that we publish. This form provides structure for consistency and transparency in reporting. For further information on Nature Portfolio policies, see our Editorial Policies and the Editorial Policy Checklist.

Statistics

For all statistical analyses, confirm that the following items are present in the figure legend, table legend, main text, or Methods section.

n/a	Confirmed	
☐	☒	The exact sample size (*n*) for each experimental group/condition, given as a discrete number and unit of measurement
☐	☒	A statement on whether measurements were taken from distinct samples or whether the same sample was measured repeatedly
☐	☒	The statistical test(s) used AND whether they are one- or two-sided<br>*Only common tests should be described solely by name; describe more complex techniques in the Methods section.*
☒	☐	A description of all covariates tested
☒	☐	A description of any assumptions or corrections, such as tests of normality and adjustment for multiple comparisons
☐	☒	A full description of the statistical parameters including central tendency (e.g. means) or other basic estimates (e.g. regression coefficient) AND variation (e.g. standard deviation) or associated estimates of uncertainty (e.g. confidence intervals)
☐	☒	For null hypothesis testing, the test statistic (e.g. *F*, *t*, *r*) with confidence intervals, effect sizes, degrees of freedom and *P* value noted<br>*Give P values as exact values whenever suitable.*
☒	☐	For Bayesian analysis, information on the choice of priors and Markov chain Monte Carlo settings
☒	☐	For hierarchical and complex designs, identification of the appropriate level for tests and full reporting of outcomes
☒	☐	Estimates of effect sizes (e.g. Cohen's *d*, Pearson's *r*), indicating how they were calculated

Our web collection on statistics for biologists contains articles on many of the points above.

Software and code

Policy information about availability of computer code

Data collection	We used commercial software, available as a package with the respective instrument, for data collection. Gels were acquired using Typhoon Phosphor Imager FLA 9500, version 1.0, Quantum CX5 Edge 18.06 and photo scanner operated with CanoScan 9000F Mark II scanner and ImageCapture v6.6(525) software. Blots were captured using Fusion FX7 Edge 18.12-5. AcquireMP (Refeyn Ltd, Version AcquireMP 2023 R1.1). GLOE-seq libraries were sequenced using NextSeq 2000.
Data analysis	Data analysis was conducted using only commercially availabe or publicly accessible software. This includes ImageJ2 (NIH, Version 2.9.0/1.53t) for the analysis of gel data; graphs and numerical data (including statistics/error bars) was analyzed and plotted by Prism10 (GraphPad, Version 10.2.3). Mass photometry measurements were analyzed using DiscoverMP software (Refeyn Ltd, Version v2023 R1.2). Multiple sequence alignment was performed with MafftWS multiple sequence alignment software version 7 and visualized with Jalview Version 2. Structural models were generated using a local version of the ColabFold v1.3 interface running iterations of the Alphafold2 v2.2.0 algorithm trained on the multi mer dataset on a local HPC equipped with NVIDIA Ampere Al00 80Go GPU cards. Custom Python script used to analyze GLOE-seq data is available on GitHub: https://github.com/Sam18-hub/CutfinderOri

For manuscripts utilizing custom algorithms or software that are central to the research but not yet described in published literature, software must be made available to editors and reviewers. We strongly encourage code deposition in a community repository (e.g. GitHub). See the Nature Portfolio guidelines for submitting code & software for further information.

Data

Policy information about availability of data

All manuscripts must include a data availability statement. This statement should provide the following information, where applicable:

- Accession codes, unique identifiers, or web links for publicly available datasets
- A description of any restrictions on data availability
- For clinical datasets or third party data, please ensure that the statement adheres to our policy

The structural models are available in ModelArchive (modelarchive.org) with the accession codes ma-m1f7g and ma-v510n for the MLH1-MLH3-EXO1 and MSH4-EXO1 complex, respectively. Movies underlying mass photometry analysis are uploaded to Dryad [https://doi.org/10.5061/dryad.6m905qgbn]. The code for data processing and analysis of GLOE-seq are available in GitHub [https://github.com/Sam18-hub/CutfinderOri]. The link will be publicly accessible upon evaluation by the Dryad team. In the meantime, please use the following temporary link: http://datadryad.org/share/iAhHwbJ7HIM3i_NKTUpYTBHoH_rB5gVBjgtKvhKy73Y
The FASTQ files generated from the sequencing of GLOE-seq libraries have been deposited in the European Nucleotide Archive (ENA) at EMBL-EBI (https://www.ebi.ac.uk/ena/browser/home) under the study accession number PRJEB88381. The raw read accession numbers are ERR14838039 and ERR14838040.
Uncropped gel images and numerical source data for graphs are included in the source data file. Protein expression constructs are available on request.

Research involving human participants, their data, or biological material

Policy information about studies with human participants or human data. See also policy information about sex, gender (identity/presentation), and sexual orientation and race, ethnicity and racism.

Reporting on sex and gender	N/A
Reporting on race, ethnicity, or other socially relevant groupings	N/A
Population characteristics	N/A
Recruitment	N/A
Ethics oversight	N/A

Note that full information on the approval of the study protocol must also be provided in the manuscript.

Field-specific reporting

Please select the one below that is the best fit for your research. If you are not sure, read the appropriate sections before making your selection.

☒ Life sciences ☐ Behavioural & social sciences ☐ Ecological, evolutionary & environmental sciences

For a reference copy of the document with all sections, see nature.com/documents/nr-reporting-summary-flat.pdf

Life sciences study design

All studies must disclose on these points even when the disclosure is negative.

Sample size	Sample size (or number of repeats) was chosen based on what is common in the field, and what was practical to do. A minimum of three independent replicates was performed for each experiment in order to add statistical analysis, when required.
Data exclusions	In general, no data were excluded unless there was a valid reason to do so, e.g. experiments with failed positive controls indicating technical problems, or when loading control indicated unequal loading that invalidated the analysis or other technical issues (broken gels, collapsed wells in gels etc.).
Replication	The experiments were repeated multiple times, as indicated in the figure legend. Oftentimes, even within a single experiment, multiple enzyme concentrations were analyzed, or samples were compared at multiple time points. This also contributes to the robustness of the data.
Randomization	Randomization is not relevant to the experiments performed in this study. This study is not sensitive to any biased analysis.
Blinding	Blinding is not relevant to the experiments performed in this study, as measurements were objectively quantified by dedicated software or simply visually presented. Furthermore, the loading order or samples on gels prevented blinding.

Reporting for specific materials, systems and methods

We require information from authors about some types of materials, experimental systems and methods used in many studies. Here, indicate whether each material, system or method listed is relevant to your study. If you are not sure if a list item applies to your research, read the appropriate section before selecting a response.

Materials & experimental systems

n/a	Involved in the study
☐	☒ Antibodies
☐	☒ Eukaryotic cell lines
☒	☐ Palaeontology and archaeology
☒	☐ Animals and other organisms
☒	☐ Clinical data
☒	☐ Dual use research of concern
☒	☐ Plants

Methods

n/a	Involved in the study
☒	☐ ChIP-seq
☒	☐ Flow cytometry
☒	☐ MRI-based neuroimaging

Antibodies

Antibodies used	The following antibodies were used for Western blotting:<br>rabbit monoclonal anti-MLH1 antibody (Abcam, ab223844, clone number EPR20522, 0.6 microgram used for immobilization in protein-interaction assays), mouse monoclonal anti-His antibody (MBL D291-3, clone OGHis, 1 microgram used for immobilization in protein-interaction assays, 1:1000 dilution for detection), rabbit polyclonal anti-FLAG (Sigma, F7425, 1:1000 dilution), mouse monoclonal anti-STREP antibody (Biorad, MCA2489, clone Strep-tag II, 1 microgram used for immobilization in protein-interaction assays), mouse anti-FLAG M2 antibody (Sigma, F3165, mouse monoclonal, Lot #SLCC4005), goat anti-mouse-HRP conjugated (Southern Biotech, 1031-05, 1:5,000, Lot #H0021-MA82E, goat polyclonal), donkey anti-rabbit-HRP conjugated (Cytiva, NA934V, 1:5,000, donkey polyclonal).
Validation	The specificity of mouse monoclonal anti-His antibody (MBL D291-3, clone OGHis) and anti-STREP antibody (Biorad, MCA2489, clone Strep-tag II) are indicated in Figure 3a and 3b respectively.<br>The specificity of rabbit polyclonal anti-FLAG (Sigma, F7425, 1:1000 dilution) antibody is indicated in Figure 2d and 3h.<br>Rabbit monoclonal anti-MLH1 antibody (Abcam, ab223844, clone number EPR20522, 0.6 microgram used for immobilization in protein-interaction assays) was validated for the following application: IP, WB, ICC/IF, Flow Cyt (Intra), IHC-P and reacts with Human samples, as stated on the manufacturer's website.<br>mouse anti-FLAG M2 antibody (Sigma, F3165, mouse monoclonal, Lot #SLCC4005) was validated for the following application: IP, WB as stated on the manufacturer's website.

Eukaryotic cell lines

Policy information about cell lines and Sex and Gender in Research

Cell line source(s)	We used Sf9 cells adapted for suspension growth, available from the cell line collection of the Institute of Molecular Cancer Research, University of Zurich.
Authentication	The cell line was not authenticated.
Mycoplasma contamination	The cell line was not tested for mycoplasma contamination.
Commonly misidentified lines<br>(See ICLAC register)	No misidentified cell lines were used.

Plants

Seed stocks	*Report on the source of all seed stocks or other plant material used. If applicable, state the seed stock centre and catalogue number. If plant specimens were collected from the field, describe the collection location, date and sampling procedures.*
Novel plant genotypes	*Describe the methods by which all novel plant genotypes were produced. This includes those generated by transgenic approaches, gene editing, chemical/radiation-based mutagenesis and hybridization. For transgenic lines, describe the transformation method, the number of independent lines analyzed and the generation upon which experiments were performed. For gene-edited lines, describe the editor used, the endogenous sequence targeted for editing, the targeting guide RNA sequence (if applicable) and how the editor was applied.*
Authentication	*Describe any authentication procedures for each seed stock used or novel genotype generated. Describe any experiments used to assess the effect of a mutation and, where applicable, how potential secondary effects (e.g. second site T-DNA insertions, mosiacism, off-target gene editing) were examined.*
